# Microvascular Fibular Flap for Traumatic Humeral Defect in an Infant: A Case Report and Literature Review

**DOI:** 10.7759/cureus.107150

**Published:** 2026-04-16

**Authors:** Alex De Carvalho, Diego T Matos, Ehab S Saleh, Ruy D Silveira Gois Neto, Iulia Dobrin, Marcus A Andrés da Silveira

**Affiliations:** 1 Hand Surgery and Microsurgery, Northeast Center of Plastic and Reconstructive Microsurgery, Aracaju, BRA; 2 Medicine, Universidade Federal de Sergipe, Aracaju, BRA; 3 Orthopedics, William Beaumont University Hospital, Royal Oak, USA; 4 Medicine, Oakland University William Beaumont School of Medicine, Rochester, USA

**Keywords:** humeral defect, infant reconstruction, pediatric microsurgery, traumatic bone loss, vascularized fibular flap

## Abstract

Microvascular fibular flaps are an established option for reconstruction of large segmental bone defects, but their use in infants following trauma is exceptionally rare. A six-month-old male infant sustained a gunshot wound causing a 5-cm humeral diaphyseal defect. Definitive reconstruction at nine months of age employed a 7-cm osteocutaneous fibular free flap with a 4 × 2 cm skin paddle. The graft was inverted and fixed intramedullary with a K-wire; microvascular anastomoses connected the peroneal artery to a proximally transposed radial artery and flap veins to superficial veins using 10-0 nylon. Early union occurred within one month. At four-year follow-up, graft hypertrophy and full limb function were observed, with only mild donor-ankle valgus. This case demonstrates that vascularized fibular transfer is feasible and effective even in infancy when performed by experienced microsurgical teams and highlights technical modifications that facilitate safe anastomosis in diminutive vessels.

## Introduction

Since the advent of microvascular bone reconstruction, the vascularized fibular flap has become a cornerstone for bridging extensive segmental bone defects, typically those exceeding 6 cm or occurring in compromised biological beds, caused by trauma, infection, neoplasia, and congenital disorders [1-4]. While alternative strategies such as non-vascularized bone grafting or distraction osteogenesis exist, they are often limited in infants by high resorption rates or the practical challenges of management. The vascularized fibular flap remains the preferred option due to its consistent pedicle anatomy, sufficient length, and unique biological potential in pediatric patients. In children, a transferred fibula can undergo significant remodeling and hypertrophy [5-7]. This process, mediated by Wolff’s law and preserved periosteal vascularity, allows the graft to eventually resemble the native bone it replaces.

Despite these advantages, microvascular transfers in infants, particularly for traumatic long-bone loss, remain extraordinary [8-10]. A focused review identified only two previous cases of vascularized fibular transfer performed in patients younger than one year, both for mandibular reconstruction following oncologic resection: Guo et al. (2008) [5] and Marques et al. (2014) [8]. To date, no instance of a long-bone reconstruction with a microvascular fibular flap has been described in an infant for traumatic defects. We report the course, technique, and four-year outcome of a nine-month-old infant who underwent humeral reconstruction with an osteocutaneous fibular free flap after a gunshot injury. This report emphasizes the technical adaptations required for successful microsurgery in infancy and documents the long-term success of graft remodeling in the youngest patient reported for this indication.

## Case presentation

**Figure 1:**
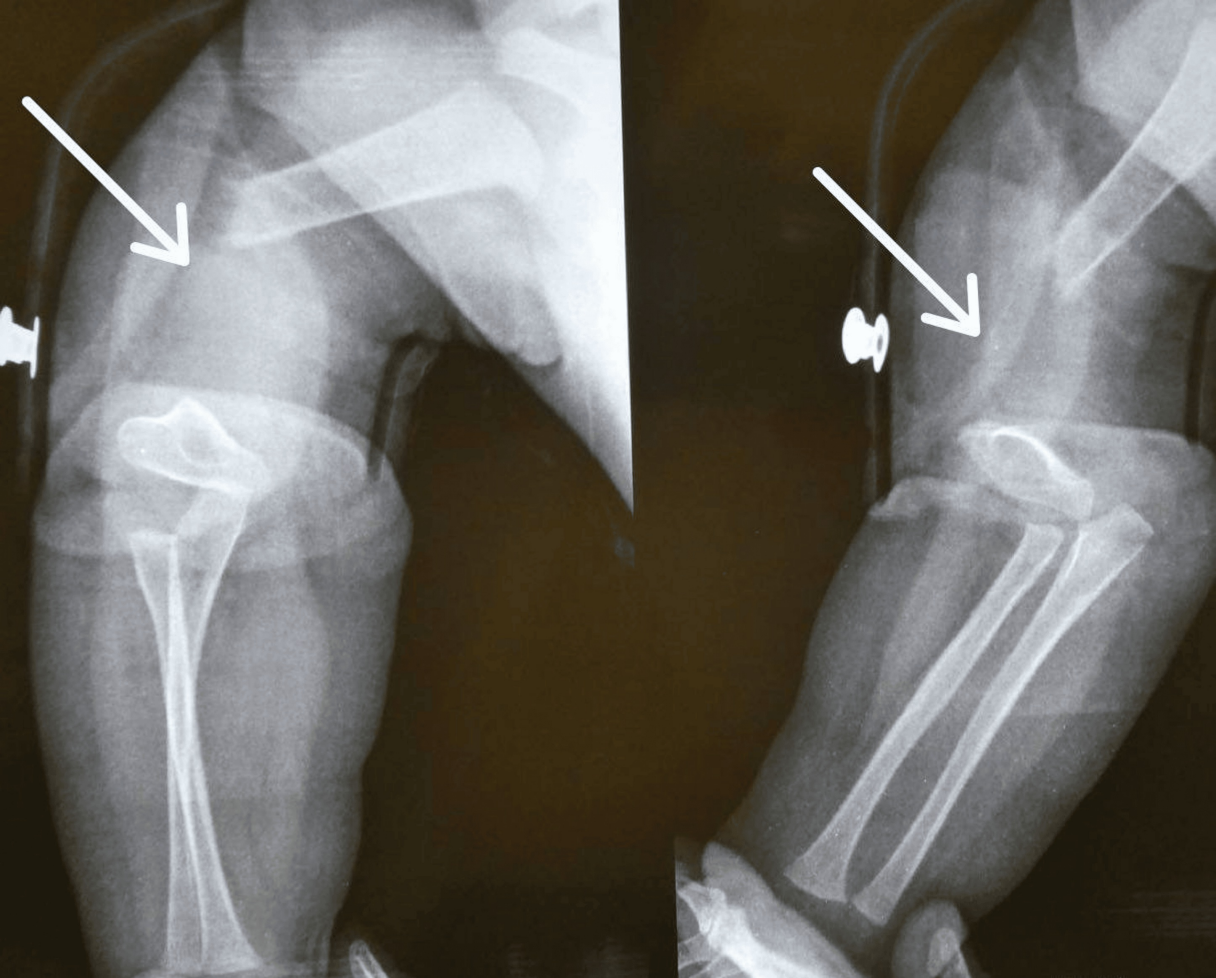
Preoperative radiograph demonstrating the segmental diaphyseal defect of the right humerus

The patient was a six‑month‑old healthy male infant at the time of injury who presented to a level I trauma center with a penetrating gunshot wound to the right arm resulting in extensive soft tissue loss and exposure of the mid‑diaphyseal humerus. Initial management included thorough surgical debridement and temporary stabilization with a laterally placed uniplanar external fixator as a damage control measure to maintain humeral length and alignment. This was supplemented by systemic antibiotics and meticulous local wound care. After soft tissue healing and clinical stabilization, the patient was referred for definitive reconstruction. On evaluation, the affected limb was shortened and flail with multiple healed scars; distal perfusion was present on Doppler, and no gross sensorimotor deficit was detected. Plain radiographs confirmed segmental loss of approximately one-third of the humeral shaft, as shown in Figure 1. Given the magnitude of the defect and the biological advantage of a vascularized transfer capable of growth and remodeling, an osteocutaneous fibular free flap was planned.

**Figure 2:**
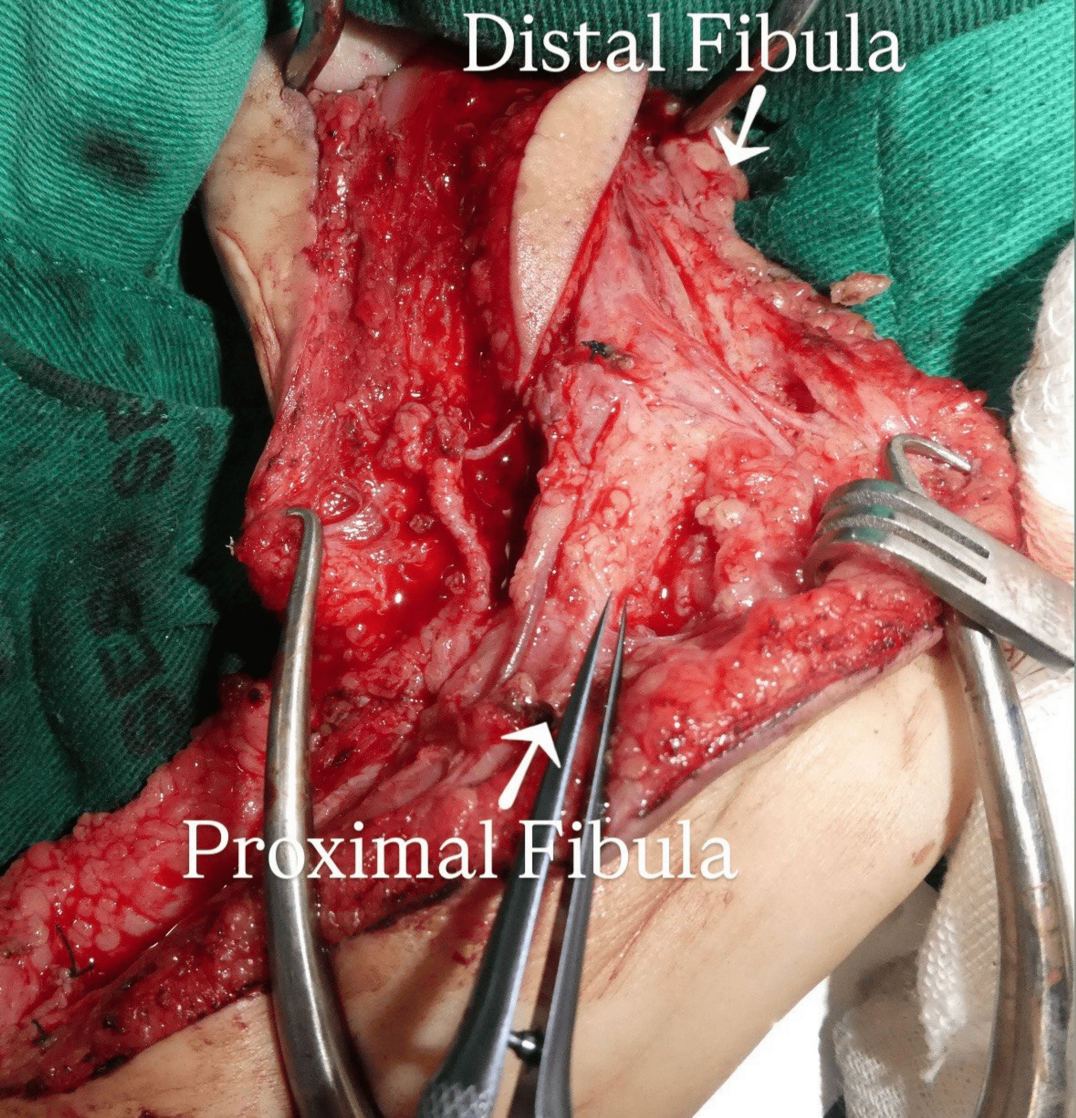
Intraoperative photograph of the harvested osteocutaneous fibular flap oriented for transfer with proximal and distal segments annotated

At nine months of age, the patient was taken to the operating room. A medial approach to the arm extending across the elbow into the mid‑forearm provided exposure to the radial artery and the superficial venous system, permitting mobilization and proximal transposition of recipient vessels. After additional debridement, the final defect measured 5 cm, as seen in Figure 2. A 7-cm osteocutaneous fibular flap with a 4 × 2 cm skin paddle was harvested from the ipsilateral lower limb, with all surgical stages performed by a single surgeon, one assistant, and one scrub nurse. To protect the distal physis and maintain ankle stability, approximately 2 cm of the distal fibular stump was preserved and stabilized to the tibia via a distal tibiofibular synostosis. The fibular graft was inverted so that its proximal segment reconstructed the distal humeral remnant and its distal segment restored the proximal diaphysis. Primary fixation was achieved with an intramedullary K-wire, which was supplemented with autologous cancellous bone graft harvested from excess segments of the fibula. This additional grafting was specifically utilized to optimize rotational stability and to ensure that the final humeral length remained symmetrical with the healthy contralateral limb, preventing over-lengthening of the reconstructed segment.

**Figure 3:**
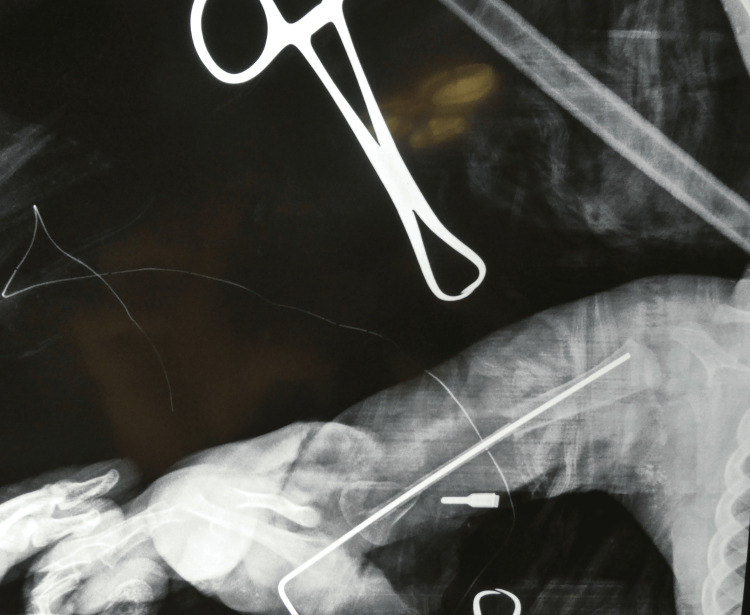
Immediate postoperative radiograph of humeral fixation with an intramedullary K-wire

Microvascular anastomoses were performed under operating microscope magnification using 10‑0 nylon in an end-to-end fashion between the peroneal artery and the proximally transposed radial artery, and venous outflow was established by anastomosing the flap veins to the mobilized superficial/comitans veins. Adequate arterial inflow and venous drainage were confirmed intraoperatively. The 8-hour 20-minute surgery was performed sequentially by a single surgeon and assistant. Recipient preparation (2 hours) and flap harvest (2 hours) utilized a 90-minute tourniquet to preserve physes and minimize ischemia. While the assistant closed the donor site, the surgeon performed graft inversion and fixation. Microvascular anastomoses required 2 hours and 10 minutes. Postoperatively, the limb was splinted, and the patient was monitored in the pediatric ICU, with the immediate postoperative radiograph seen in Figure 3. The patient was discharged on day 7 in stable condition.

**Figure 4:**
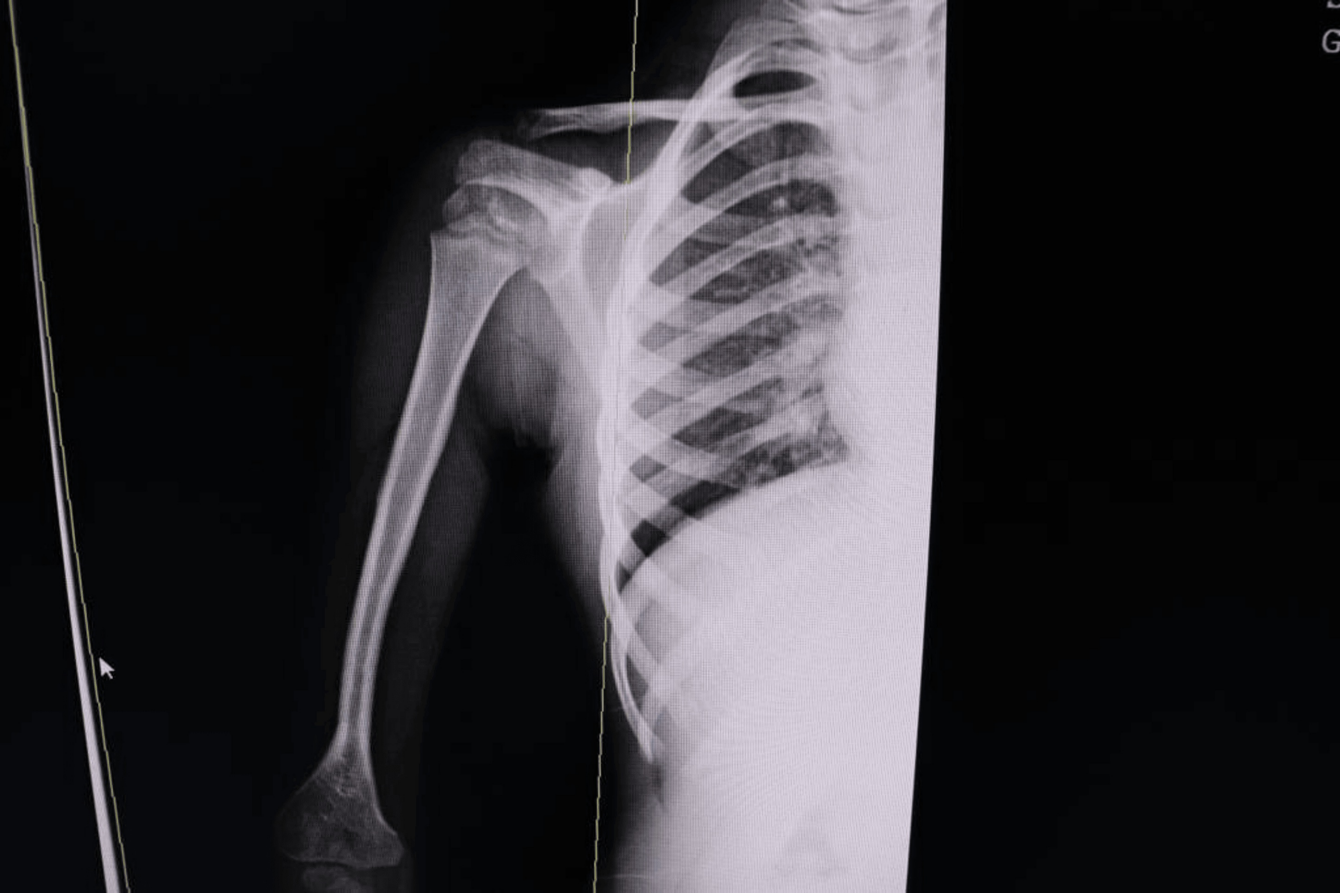
Radiograph at four years postoperatively demonstrating graft hypertrophy and remodeling

Early radiographs demonstrated progressive union, and by one month, there was sufficient consolidation to permit removal of the intramedullary K‑wire. The postoperative course was notable for a Clavien-Dindo I minor wound dehiscence at the elbow that healed with local care and subsequently required Z‑plasty to correct scar contracture. There was no flap loss; no deep infection, defined as a condition requiring intravenous antibiotics or surgical intervention; and no donor site failure. At four years of follow‑up, the child exhibited symmetrical shoulder and elbow function compared with the contralateral limb, radiographic evidence of graft hypertrophy and remodeling consistent with adaptive growth, as shown in Figure 4. A mild valgus angulation of the donor ankle, approximately 8°, was present but produced no functional impairment. Objective functional measures, such as goniometric range of motion values and standardized pediatric scoring systems, were not available for this report but should be included in future series to strengthen outcome assessment.

## Discussion

Microvascular reconstruction in infancy presents formidable technical challenges. Small vessel caliber, fragile tissues, and narrow operative fields increase the risk of thrombosis and complicate fixation. In this case, inversion of the fibular graft and transposition of the radial artery and superficial veins were critical adaptations, simplifying the procedure and permitting tension-free, end-to-end anastomoses without vein grafts.

Beyond the technical success, this report holds particular significance in the medical literature. A focused review identified only two previous cases of vascularized fibular transfer performed in patients younger than one year, both for mandibular reconstruction following oncologic resection-Guo et al., 2008 and Marques et al., 2014. No prior instance of a long-bone reconstruction with a microvascular fibular flap in an infant was identified in our literature search. Thus, this case represents, to our knowledge, the youngest patient ever reported to undergo this procedure for a traumatic humeral defect.

The successful outcome emphasizes that, with appropriate expertise and vessel planning, microvascular bone transfer can be safely achieved even in early infancy, combining mechanical stability with the biological potential for hypertrophy and remodeling, a process mediated by Wolff’s law and the preserved periosteal vascularity of the transfer. Donor-site stabilization with a distal tibiofibular synostosis mitigated ankle instability, and four-year follow-up demonstrated only a mild, asymptomatic valgus deformity of approximately 8°. Although this single case cannot define universal indications, it reinforces the viability of vascularized bone transfer in selected infants, warranting the establishment of prospective multicenter registries with standardized functional and radiologic endpoints to delineate long-term growth effects and refine operative technique.

This case report has several limitations, including its nature as a single-institution experience and the potential for reporter bias, as the four-year follow-up was conducted solely by the operating surgeon during routine outpatient visits. Additionally, the lack of objective, validated pediatric functional outcome measures limits the ability to quantify recovery beyond clinical observation. Alternative reconstructive options were considered but ultimately deemed less favorable. Non-vascularized bone grafting carries a high risk of resorption and non-union in a 5-cm segmental defect within a traumatic bed, and it lacks the inherent growth potential of a vascularized transfer. Distraction osteogenesis was also discounted due to the significant challenges associated with pin-site management, infection risk, and the prolonged treatment duration required in a nine-month-old infant. A vascularized fibular flap was selected because it provides immediate biological activity and superior structural stability, which are essential for hypertrophy and long-term remodeling as the patient grows.

## Conclusions

Vascularized fibular transfer is a feasible and effective option for segmental long-bone reconstruction in infancy when performed by a specialized microsurgical team. Technical modifications (graft inversion and radial artery transposition) facilitate anastomosis in very small vessels, which in this case measured approximately 0.8 mm in diameter. This case extends the boundaries of pediatric microsurgery, documenting the first successful long-bone fibular transfer in a nine-month-old infant after trauma.

## Appendices

Ethical statement

Written informed consent for publication of clinical details and anonymized images was obtained from the patient’s legal guardian. The Institutional Ethics Committee of the Federal University of Sergipe confirmed that formal approval for single case reports was not required.

Conflicts of interest

The authors declare no conflicts of interest.

Funding

No external funding was received.

Acknowledgments

The authors acknowledge the operating room and pediatric ICU teams for their support in patient care.
